# State Board of Health

**Published:** 1875-09

**Authors:** Joseph P. Logan

**Affiliations:** Chairman of Special Committee.; Atlanta, Ga.


WE ARE REQUESTED by Dr. Logan, in behalf of the Board of Health, to bring to the attention of our Georgia readers the following request, which we trust will meet with a prompt and hearty response from those possessing the desired information:

ATLANTA, GA., August 20th, 1875.

In behalf of the special committee of the State Board of Health, upon "the most effectual means of preventing small pox, including specially a more thorough vaccination, and the isolation of those effected with the disease," I shall be obliged for any reports of facts in regard to the occurrence of this disease, with statistics as to number of cases, whether supposed to be vaccinated, and rate of mortality. It is desirable that these reports should not be delayed longer than the 15th of September, 1875.

JOSEPH P. LOGAN,
Chairman of Special Committee.

